# “Mending fractured personalities”: A photography-based cultural study of recovery from mental distress in Romania

**DOI:** 10.1177/13634615221119373

**Published:** 2022-08-29

**Authors:** Lucian Hadrian Milasan

**Affiliations:** Nottingham Trent University, UK

**Keywords:** culture, mental health, photo-elicitation, photography, recovery, Romania

## Abstract

This study explored the lived experiences and meanings of recovery from mental distress from the perspective of mental health service users in Romania, along with investigating cultural particularities of recovery in this country. Research in this area is essential in the context of Romania's mental health reform marked by a transition from institutionalised mental health services to a recovery-based approach, and profound social and economic changes during the post-communist era. Subscribing to the recovery framework, this study employed a qualitative phenomenological design involving 15 adults with mental health problems purposively recruited from a community day centre in Romania. The phenomenological background was enriched with elements of participatory photography to elicit subjective experiences and meanings of recovery. The outcome of this study was a better understanding of recovery in Romanian adults living with mental distress, as a complex and multi-layered phenomenon. Three key themes were identified through Interpretative Phenomenological Analysis: awakening, healing, and reconstructing life. The findings add to the current recovery models by showing that recovery cannot be fully understood unless situated in a socio-political, cultural, and historical context. Implications for mental health practice in Romania are discussed and directions for future research are recommended.

## Introduction

Recovery represents the cornerstone of mental health services by guiding their reformation worldwide (Slade & Wallace, 2017). Within this context, understanding recovery is essential, but is not always a straightforward process. This is due to tensions between recovery conceptualised as a biomedical phenomenon and recovery as a personal experience determined by psycho-social factors (Macpherson et al., 2016). On the one hand, recovery is traditionally understood as an outcome consisting of remission of symptoms following pharmacological treatments (Deacon, 2013). On the other hand, service user-led research points towards recovery as a holistic process of achieving a “personally acceptable quality of life” (Law & Morrison, 2014, p. 40), beyond symptom management and compliance with pharmacological treatments. This conceptualisation is in line with the seminal definition of recovery advanced by Anthony (1993, p. 21) as “a deeply personal, unique process of changing one's attitudes, values, feelings, goals, skills and/or roles […] a way of living a satisfying, hopeful, and contributing life even with limitations caused by the illness.” However, even service users’ perspective on recovery is nuanced, with increasing criticism of the language of recovery and activism towards emplacing recovery in the context of social justice (Recovery in the Bin, 2017). On the same note, intersectional approaches examining socio-political and cultural factors of recovery have been developed, providing a social justice framework of mental health recovery (Morrow & Weisser, 2012).

While debates on this topic remain heated, with some authors emphasising the complementarity of clinical and personal recovery (Slade, 2009), it is widely acknowledged that the cultural understanding of recovery is limited (Rudnick, 2012; Slade et al., 2014). As a result, the applicability of recovery models, (e.g., the Tidal Model (Barker & Buchanan-Barker, 2005), the Strengths Model (Rapp & Goscha, 2012), the CHIME recovery framework (Leamy et al., 2011), and Whitley & Drake's (2010) dimensional model), in various cultural contexts remains uncertain.

Mental distress is a complex phenomenon interweaved in the cultural fabric of society (Chiu et al., 2010; Tse et al., 2012). Culture is understood here as values, beliefs, customs, and ways of life of people sharing a common origin and historical background (Phelps & Nadim, 2014). It also includes “the derivatives of experience […] learned or created by the individuals of a population, including those images or encodements and their interpretations (meanings) transmitted from past generations, from contemporaries, or formed by individuals themselves” (Schwartz, 1992, p. 324). It is claimed that culture influences individuals’ perception of mental distress and how this is understood, accepted, and treated (Çam & Uğuryol, 2019). Stigma and discrimination in relation to mental distress also are believed to be determined culturally (Bracke et al., 2019). Additionally, processes of recovery and meanings attributed to this phenomenon are different in various cultural contexts (Boucher et al., 2019; Eltaiba & Harries, 2015; Gopal & Henderson, 2015; Lapsley et al., 2002; Ng et al., 2008; Thara, 2012).

Therefore, exploring recovery in relation to cultural aspects may provide an enhanced understanding of the complexity of this phenomenon, and suggest potential avenues to support recovery in ways that are culturally appropriate and acceptable. Furthermore, this could inform the current recovery frameworks criticised for giving insufficient attention to cultural contexts of recovery (Price-Robertson et al., 2016). On this note, O’Hagan (2001) stresses the importance of acknowledging “cultural diversity and a connection to one's own culture as key to recovery” (p. 2).

The extent to which cultural factors influence views on recovery in mental health has not yet been researched in Romania. The term “recovery” does not have an accurate translation in the Romanian language; the closest term is “recuperare” (“recuperation”), strongly linked to physical illnesses. Such connotations may result from the predominance of the medical model that continues to guide mental health services in this country (Friedman, 2016). Moreover, it is claimed that person-centred service delivery models in Romania are implemented without a prior understanding of the service users’ view on recovery, relying mainly on imported recovery measures from English-speaking countries (Shields-Zeeman et al., 2020). This may be problematic for the reformation of mental health services that need to factor in cultural and historical particularities of this country, and its transition from institutionalised mental health care delivered in large-scale psychiatric hospitals, to community-based services (Andronic & Andronic, 2017; WHO, 2017).

Another novel dimension added by this study to the mental health research landscape in Romania is the use of photography-based methods shown to facilitate the exploration of spatial, social, and affective dimensions of the lived experience of recovery (Milasan, 2022). Methodologically, this adds to an increasing body of international research embedding visual artefacts of recovery to deepen the understanding of this phenomenon (Cabassa et al., 2013; Mizock et al., 2014; Sitvast, 2015; Vansteenkiste et al., 2021). Combined with a lived experience framework (Ellis & Flaherty, 1992), photography was employed in this study to investigate the subjective perspectives on recovery as expressed by individuals situated in the physical, historical, and political context of Romania.

## Aim

Within this context, the aim of this study was to explore lived experiences and meanings of recovery from mental distress from the stance of mental health service users in Romania. Additionally, it investigated potential cultural, socio-economic, and historical particularities of recovery, a topic that is currently under-researched in this country.

## Method

### Recruitment

Fifteen participants (seven females and eight males, age 28 to 63) from different socio-economic, ethnic, and educational backgrounds were purposively recruited from a mental health community day centre in Romania in May 2019. They reported various experiences of psychological distress, predominantly psychosis, and were following psychiatric treatments at the time of conducting the research. Participants provided informed consent (including the dissemination of their photographs) and completed a questionnaire providing background demographic, social, and medical information (summarised in Table 1). The study was granted ethical approval from the Faculty of Health and Medicine Research Ethics Committee at Lancaster University (FHMREC18047, 25 February 2019), and permission from the host organisation in Romania (Estuar Foundation).

**Table 1.** Socio-demographic and medical characteristics of the research participants.

**Number of participants**	15
Male	8	(53%)
Female	7	(47%)
**Average age of participants**	43
Lowest age	28
Highest age	63
**Ethnicity**	
Romanian	12	(80%)
Hungarian	3	(20%)
**Education**	
Junior school	2	(13%)
Vocational studies	3	(20%)
College	6	(40%)
University	4	(27%)
**Employment status**	
Unemployed	14	(93%)
Employed	1	(7%)
**Marital status**	
Single	10	(66%)
Married	1	(7%)
Divorced	3	(20%)
In a relationship	1	(7%)
**Mental health problems (diagnosis)**	
Schizophrenia (psychosis)	13	(87%)
Depression and anxiety	2	(13%)
**Average age when first experienced mental distress**	27
**Medical treatment**	
Antipsychotics	13	(87%)
Antidepressants	2	(13%)
**Non-medical treatment (counselling or psychotherapy)**	
Previously accessed	5	(33%)
Never accessed	10	(67%)
**Average years of accessing mental health services at the community day centre**	9
Minimum	1
Maximum	21

### Data collection

Textual and visual data required to illuminate experiences and meanings of recovery were collected in two stages: (i) four weekly photography workshops (n  =  11 participants), followed by (ii) semi-structured photo-elicitation interviews (n  =  12). Data were collected over a period of eight weeks (June to August 2019), during which time some of the research participants took part in both photography workshops and interviews (n  =  8), or only interviews (n  =  4) or workshops (n  =  3), depending on their preferences and availability. This approach facilitated the broad exploration of the research topic during the first stage that informed the development of the interview guide, and created further directions for research during the second phase. As a result, during the interviews, key areas of recovery developed during the photography workshops were approached in more depth with the research participants, and then clarified and reflected upon.

Participants were provided with digital photo cameras and instructions on how to use them, and were invited to take pictures of objects or people (with written consent) that were representative of their experience of recovery. Images were then brought to the photography workshops and individual interviews, and three to five of the images were selected by participants together with the researcher and critically discussed.

To enhance the research quality and ensure participant engagement in the development of recovery themes, a reflection and feedback group was organised at the end of the series of photography workshops. This additional activity gave the researcher and research participants the opportunity to address any misinterpretations or disagreements in relation to, but also elaborate on, their initial understanding and meanings of recovery. All research activities were audio recorded, transcribed, and translated from Romanian into English prior to being analysed.

### Data analysis

This study subscribes to a constructivist/interpretivist paradigm centred around social construction of meanings through symbolic interaction and the pluralistic nature of reality (Denzin, 2004). Hence, the method chosen was Interpretative Phenomenological Analysis (IPA; Smith et al., 2009), and the steps followed iteratively by the researcher were: (1) reading and rereading the original transcripts and checking for accuracy; (2) initial noting and labelling i.e., breaking down textual and visual data into discrete entities; (3) identification of key themes; (4) searching for connections between key themes; (5) moving onto the next case; and (6) looking for patterns across cases. Participants’ interpretation of their own experiences of distress and recovery elicited through photographs informed the subsequent stages of the data analysis conducted by the researcher, aided by Atlas.ti® and mind-mapping. Visual data were interpreted together with the research participants during the workshops and interviews to extract the meanings they attached to recovery in their photographs. This approach was preferred to an iconographic analysis which was shown to potentially lead to interpretation bias, hence the need to involve research participants more actively in the process of analysing photographic content (Mizock et al., 2014).

Particular attention was given to commonalities, but also particularities, of participants’ perspectives on recovery, in line with IPA's idiographic approach. Finally, the principle of double hermeneutics (Nørreklit, 2006) enhanced the analytical value of this study by guiding the researcher's interpretation of how participants made sense of their recovery.

### Findings

Following IPA of the four photography workshops and twelve interview transcripts (totalling 22 h of audio-recording and 89 images selected from a total of 1,118), three key interlinked recovery themes – awakening, healing, and reconstructing life – presented below, were identified along with their corresponding subthemes in the verbal and visual narratives of the research participants (referred to in this section by their pseudonyms of choice).

### Awakening

Conceptualised initially as an act of becoming aware of life beyond the limitations associated with mental distress, awakening was revealed as a nuanced dimension of recovery from overcoming the side-effects of psychiatric treatments to more symbolic aspects such as consciousness, self-awareness, and freedom.

#### Overcoming sedation

Meanings of recovery were critically discussed by participants in the context of a psychiatric system described as being over-medicalised and focused almost exclusively on service users’ compliance with medication. Ironically, psychiatric treatments often caused participants more distress than their own mental health problems:

I was drugged up and I was feeling really unwell. And they changed my medication every so often. I was feeling increasingly anxious because I was taking medication for something that probably wasn’t there. This is because I was given loads of misdiagnoses. (Daniela, Interview)

Hence, participants understood recovery as a process consisting partly of overcoming a state of sedation from strong and debilitating psychiatric treatments. Narratives of helplessness and incapacitation following high doses of medication revealed the loss of varied and productive routines.

For example, medication interfered with the day-to-day life of Rodion, who experienced severe Parkinson-like side-effects:

It was frightening because for one month and a half I was sitting like those old people […] shaking and lacking concentration because it [the medication] diminishes your mental faculties. The injection [anti-psychotic] stopped me from doing things like playing games on computer, riding the bike, sports … I just couldn’t do that [anymore]. (Rodion, Interview)

Another participant talked about the way medication impaired her cognition, physical and social mobility, and her promising life prospects that she developed as a hard-working student:

Things weren’t that good after I started taking medication. [I was feeling] worse and worse, worse and worse, until I was given a diagnosis, and then another one, more severe. And I was taking more and more medication, and I wasn’t able to do anything. (Agnes, Workshop 2)

Memory recall was also affected by high doses of medication in some participants who were subjected (mostly involuntarily) to multiple sessions of electroconvulsive therapy (ECT). Ioana (Workshop 4) described such experiences as the “dark side” of her existence and, consequently, conceptualised recovery as a “return to light,” a recurrent metaphor in participants’ photographs. On the same note, Robert reminisces:

I remember going back home a little bit sleepy and lacking life up to a point when I reduced my medication. And, increasingly, I felt the need to take part in activities at the day centre. I gradually felt an awakening inside, that I can do this and even more. (Robert, Interview)

**Figure 1.**
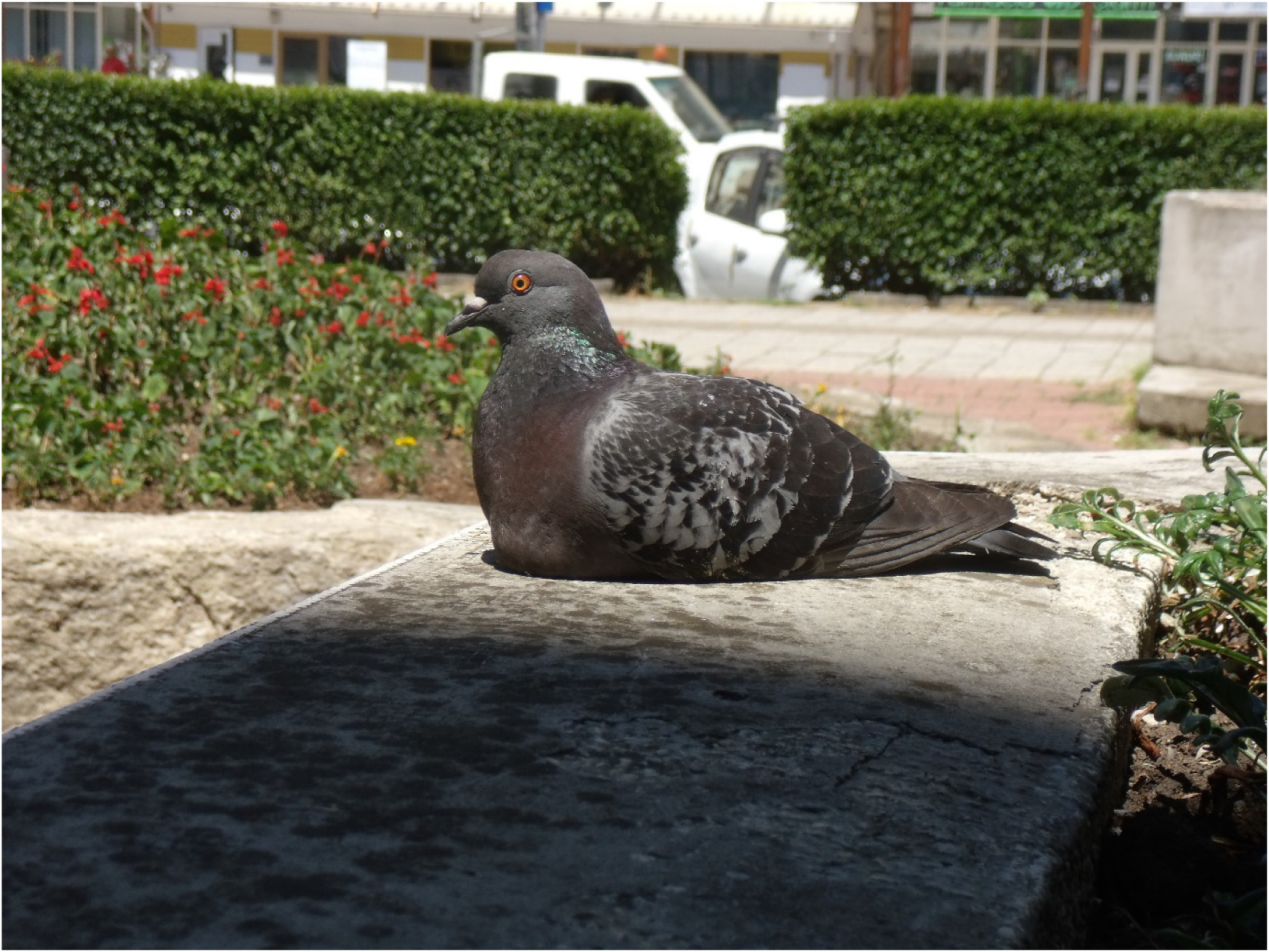
“This is a pigeon – floppy like me […] These pills make us sleepy.” (Deirdre, Workshop 2).

Photography aided participants’ description of increased sensorial experiences and awareness of their surroundings, which accompanied their process of recovery (Figure 1).

#### Emergence of self beyond illness

The journey through darkness and mental distress resulted for some participants in a process of self-discovery emerging from a renewed sense of being in the world, feeling and thinking beyond the fluid boundaries of illness: “I gradually started to find myself, to feel that I am useful, determined, and able to make decisions for myself and my family” (Iaco, Interview). For Robert, the process of self-discovery took place in the context of activities at the day centre, a milestone to his recovery. Here, peer support helped him realise the importance of being aware of self and his own potential through meaningful exchanges with others:

Here, at the day centre, I learned to pick myself up. You come here and you are part of it, you exist, you identify yourself with it [the day centre], you give them [other service users and staff] a part of you. (Robert, Interview)

**Figure 2.**
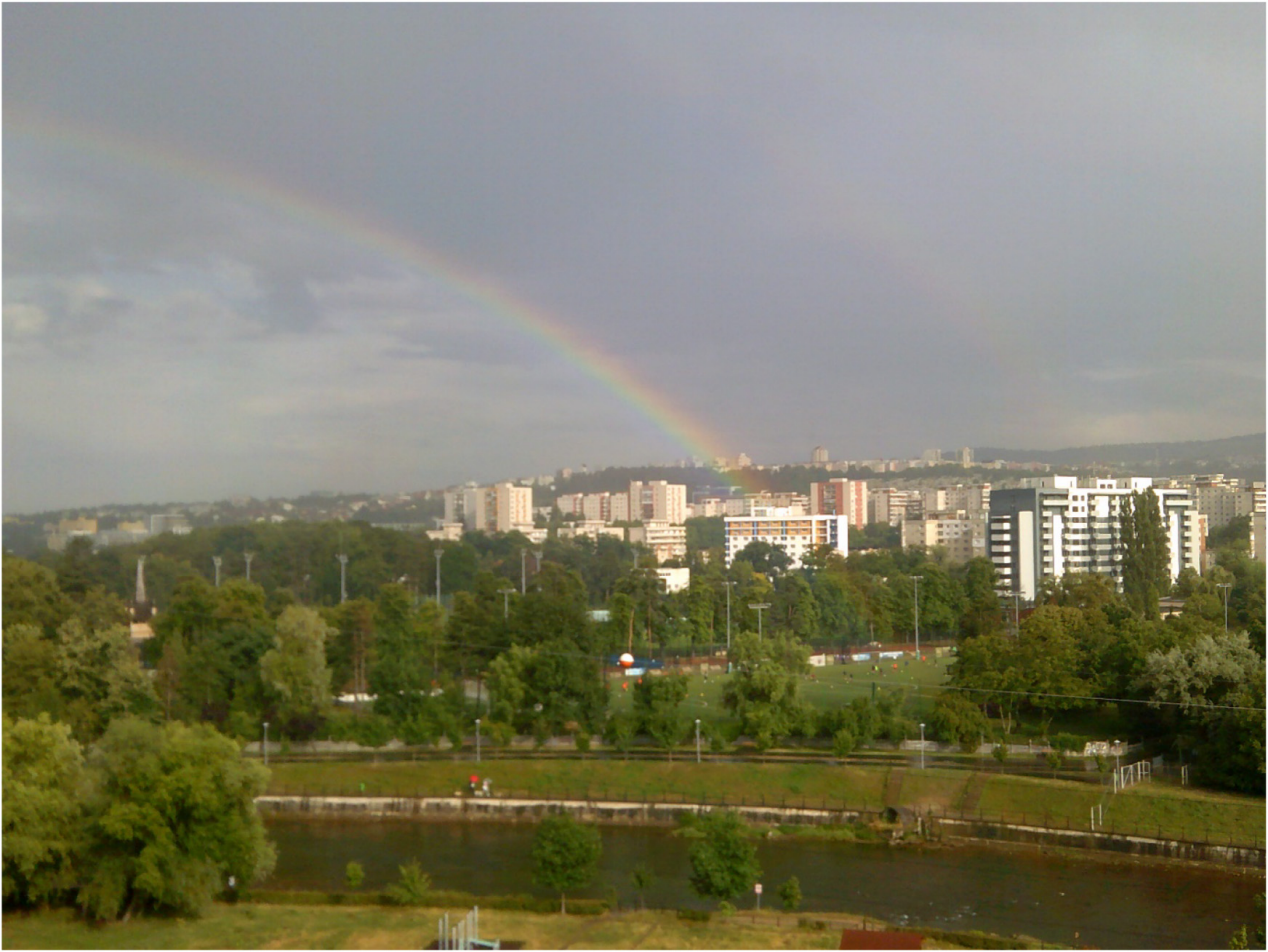
“After rainy and stormy days, there's an awakening in my soul that gives me wings. It makes me feel alive.” (Robert, Interview).

For some participants, self was defined by feelings more than actions. In this context, recovery was centred on regaining their ability to nurture positive feelings and “take in the beauty of the inner and the outer world” (Agnes, Workshop 2). This process was facilitated by the photographic expression of emotions through metaphors that frequently included natural elements (e.g., forests, parks, gardens, skies, bodies of water, etc.) that elicited deep meanings of recovery in participants’ narratives (as illustrated in Figure 2).

One participant further explained that “recovery means to keep certain human qualities. Because many mentally ill people are like bodies maintained and kept alive with medication” (Rodion, Workshop 3). Wisdom, patience, courage to carry on, dignity, self-respect, will power, perseverance, self-motivation, and love were only some of the human traits that the research participants deemed as indispensable to recovery. This existential dimension was explored by the research participants guided by self-acceptance, self-worth, and coming to terms with their renewed identities: “Every life, no matter how much it deviates from the norm, is valuable. I mean, even if we have a mental illness, we have a life and we have to enjoy it” (Deirdre, Workshop 2).

#### Liberation

Recovery as liberation was understood by participants on an individual level, as an emerging sense of freedom from the chains of illness; and on a socio-political level, as liberation from societal constraints. In both instances, this process was imminently associated with taking ownership of one's life and constructing one’s own vision of the world in spite of still experiencing signs of distress: “It doesn’t matter how ill or schizophrenic you are. If you feel some freedom, you can concentrate better on the things that need to be done [to recover]” (Iaco, Interview).

On a personal level, for Emilian (Interview), regaining a sense of freedom was related to a lessening in his hearing of voices which previously had dictated his thinking and behaviour, but also liberation from the constraints of a conflictual relationship with his father that limited his choices in life. For others (Ioana, Rodion), personal freedom was sought in relation to the psychiatric system with its prescriptive and involuntary treatments and the sense of being controlled and reduced to receptacles of psychiatric medication: “There is freedom if you know how to see it. But you must go on the other side of the bars. There are always bars … sometimes useful for protecting people, but useless if people are in search for something” (Rodion, Interview). On a socio-political level, this subtheme was placed by older participants in the context of oppression and inhumane political and psychiatric practices during the communist era in Romania that severely impacted on their psychological wellbeing:

I didn’t consider myself ill. I became paranoid because I saw people being taken away in an ambulance [by the secret police i.e., Securitate]. And that's how I was admitted. They [doctors] told me that I won’t be sectioned, but they shut the doors behind, and I was sectioned. (Rodion, Interview)

I was admitted [to a psychiatric hospital] in ‘88. I was sectioned and they gave me electroshocks, about eighteen sessions, to help me forget because I started to tell them what was going on [i.e., being followed by the secret police (Securitate) due to taking part in anti-communist protests]. (Iaco, Interview)

For younger participants, the current challenges of corruption, poverty, and unemployment in this country were more prominent, with an impact on their levels of distress:

If I had an opportunity, I would leave [the country] and never come back. I simply don’t like what's going on in this country. There is corruption at all levels and things never get better in this country. […] I have two pensions: one for handicap 763 lei [£150] and the other one is 375 lei [£70]. I can barely manage. (Ioana, Interview)

**Figure 3.**
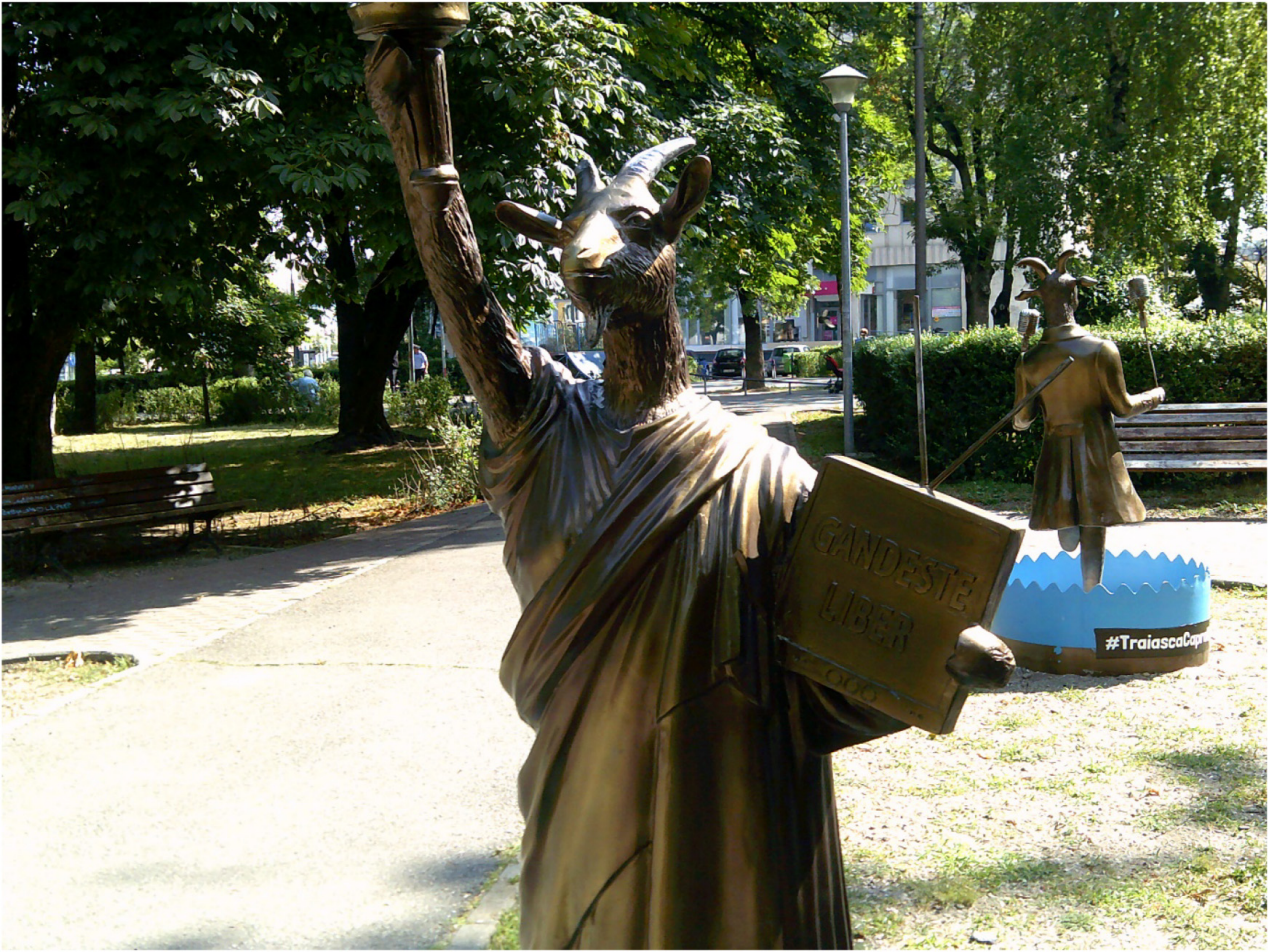
“To recover means to think freely. No one tells you what and how to think. You can make choices and do things the way you want.” (Emilian, Interview).

For some participants, becoming active citizens and exercising their right to vote to change the political status quo at the time of conducting the research was identified as part of recovering their welfare and the destiny of a country facing major economic and political obstacles. Freedom of thinking and freedom of choice also emerged from the narratives of some participants in relation to the restoration of their citizenship (Figure 3).

Less frequently, recovery as liberation was described by some participants as a collective and political act of self-determination, with mental health service users holding latent powers to regain their voices muted during decades of oppression during communism: “We are talking about an army of foot-soldiers, so it is not only an individual. It is about you as a group to take steps in illness and life” (Deirdre, Workshop 3); “… to make our choices, our decisions, when to say ‘no’ and when to say ‘yes’” (Robert, Workshop 3).

### Healing

The term “healing” was frequently used by research participants to describe their recovery from mental distress. The meanings associated with “healing” were multiple and complex, in line with participants’ various lived experiences: medical, psychosocial, and spiritual.

#### Medical healing

Medical treatments were common to participants’ experience of recovery that was equivalent for some with a reduction of symptoms: “Recovery is about not hearing voices anymore […] They have been decreasing in intensity since I got ill eight years ago. Before the treatment, they were there non-stop … now, only sometimes” (Emilian, Interview).

**Figure 4.**
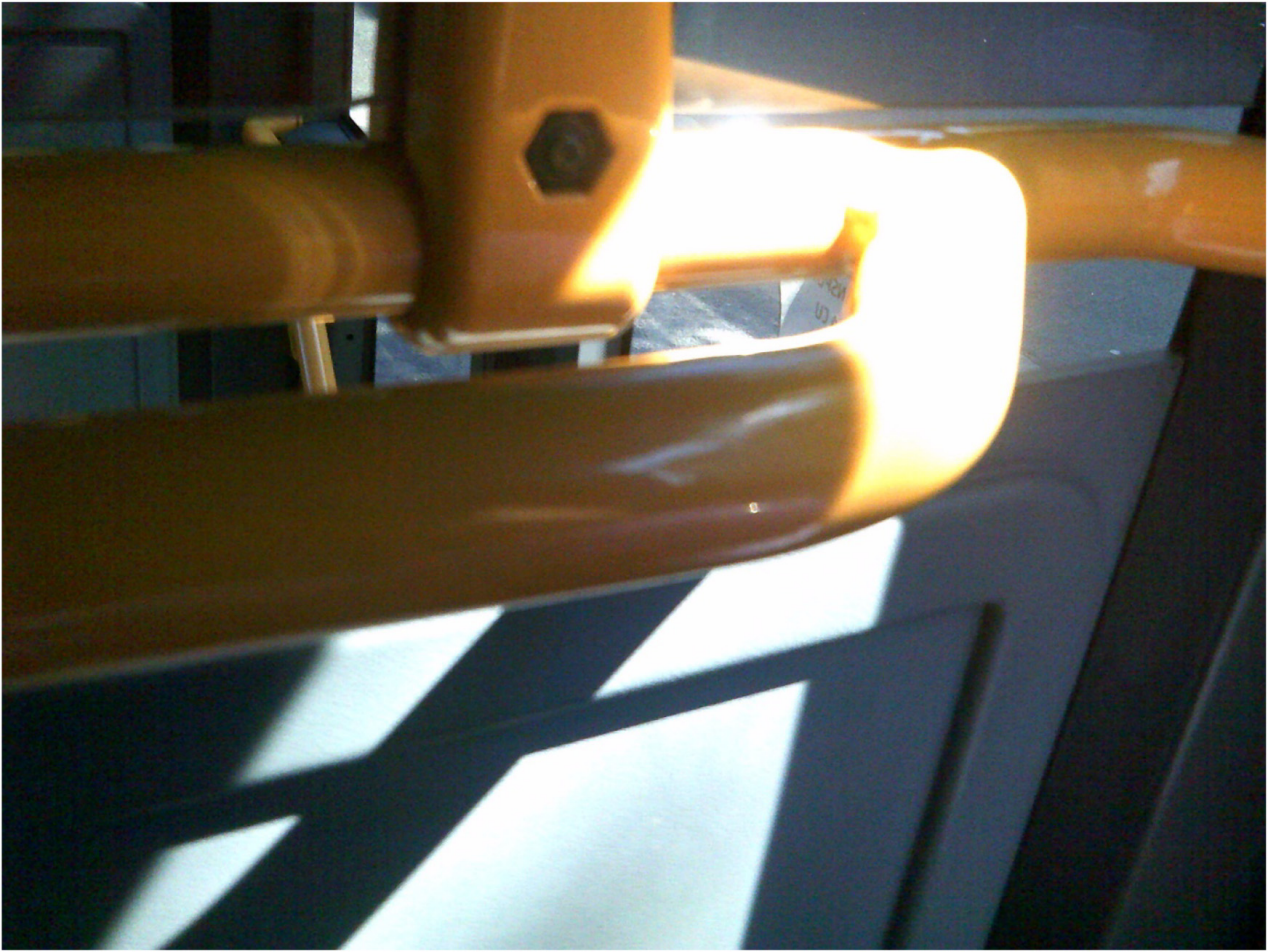
“She [the psychiatrist] was the only one who stopped by my bed and asked me face-to-face ‘How are you feeling?’ […] It was the first person I trusted […] I was feeling better emotionally knowing that someone's there for me.” (Daniela, Interview).

Medication appeared as a way of normalising life and, therefore, was key to recovery: “There is recovery from this illness. If you take your treatment and comply, you can live a normal life” (Ioana, Workshop 2). Beneficial effects of medication such as relaxation, decreased anxiety and stress, and improved sleep hygiene were identified by some participants, while others talked about distressful and burdensome side-effects of the medical treatments. Participants’ experiences of hospitalisation were also diverse, with some emphasising the importance of timely medical support when in distress. In this context, recovery was sometimes supported by encounters with highly competent and compassionate mental health professionals who inspired trust, stability, and support (Figure 4).

However, such narratives were uncommon as most of the participants suggested that medical perspectives were limited with regards to their subjective experience of distress. This may explain why some participants did not consider themselves “healed” despite complying with medication and experiencing a reduction of symptoms: “I’m quite far from being recovered, although the only treatment I take at the moment is an injection” (Raul, Interview). As a result, participants’ degree of compliance with psychiatric treatments varied widely, from taking the medication almost religiously, to those reluctant to take it, or even discontinuing their treatment, mainly because of the side-effects and the lack of trust in the psychiatric system.

#### Psychological healing

**Figure 5.**
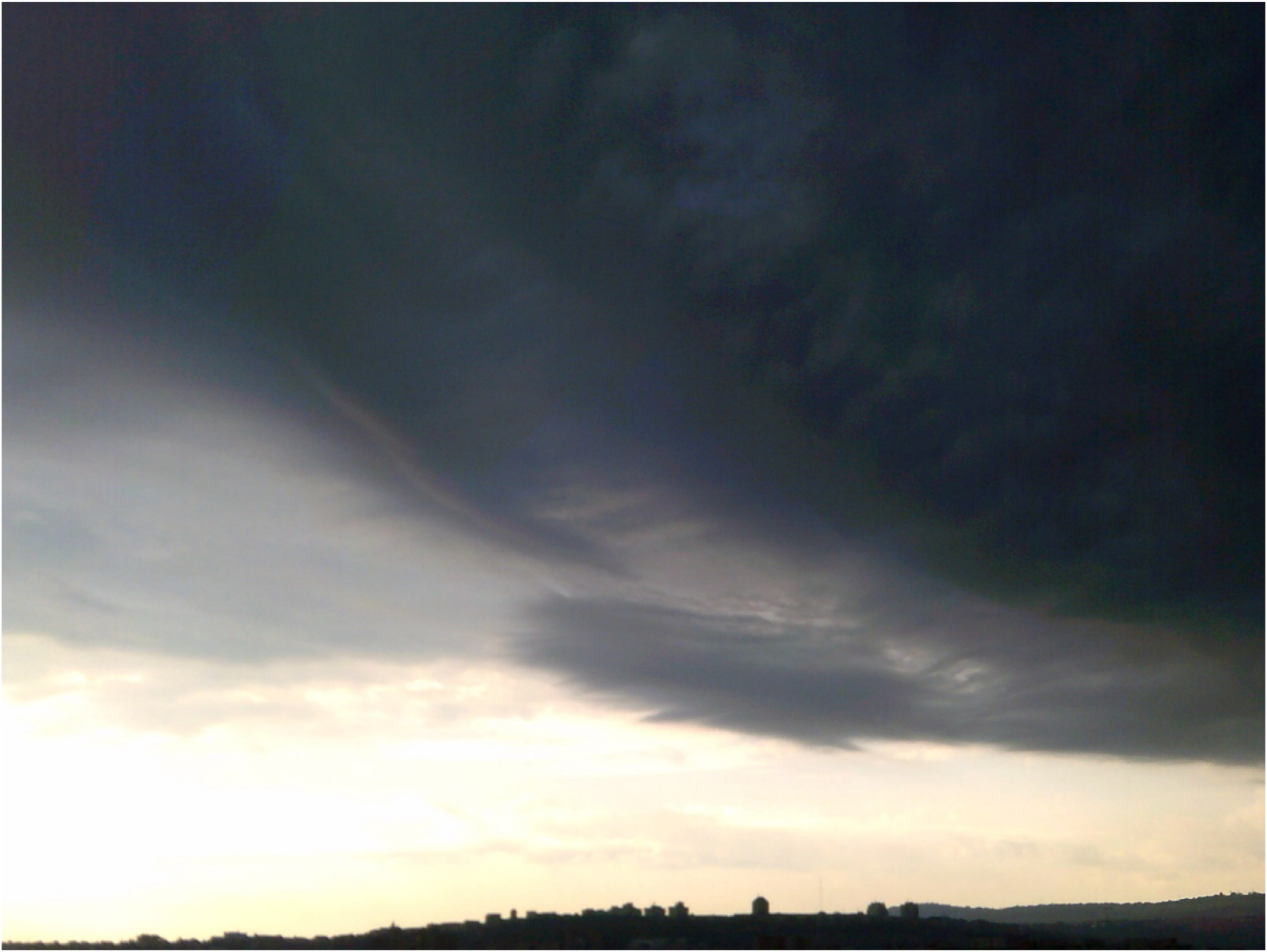
“I have a worry, a fear, when the dark is coming.” (Robert, Workshop 2).

Participants’ narratives revealed mental distress as a psychological experience characterised by a deterioration of thoughts and feelings, frequently expressed through verbal (e.g., “wounds of the soul”) and visual metaphors (e.g., “dark thoughts,” illustrated in Figure 5).

**Figure 6.**
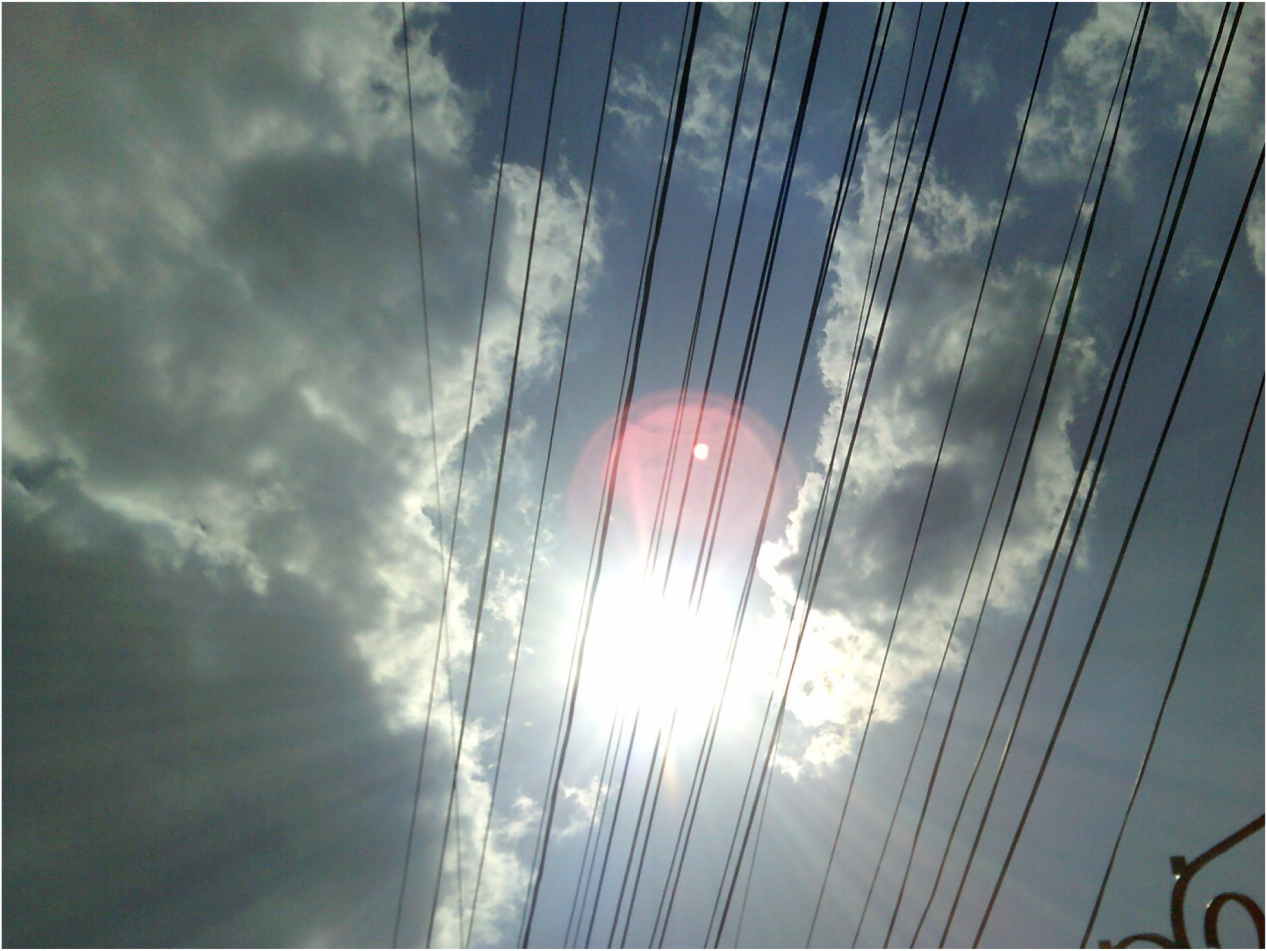
“The clouds mean sadness or depression. And the sun means something bright that brings light to that sadness.” (Agnes, Workshop 2).

Exploring experiences and meanings attributed by participants to their distress was essential for understanding their making sense of recovery as psychological “healing,” also referred to as “cleansing,” “sieving,” “purification,” or “transition” from negative to positive feelings and cognitions (Figure 6).

Participants’ understanding of recovery as healing of a fragmented mind or “mending fractured personalities” (Deirdre, Workshop 3) appeared to be influenced by external knowledge and explanations learned from their psychiatrists: “They [doctors] explained to me that our mind is split in many parts that we have to bring together” (Emilian, Interview). As a result, participants reiterated the importance of psychological treatments that were not easily accessible or affordable to them:

I wish I could have had psychotherapy, but I didn’t have the chance. I didn’t know where I can access therapy. Psychotherapy in Romania also depends on costs. I didn’t have the [financial] means, but it would have been good for me. (Raul, Interview)

This may explain why self-help and alternative therapies (e.g., arts-based, photography, bibliotherapy, and sports) were essential to some participants’ recovery: “Photography helps us capture the beautiful aspects of life. To discover that, despite seeing the world in dark colours, there is sunshine” (Deirdre, Workshop 4). Furthermore, participants revealed the medical connotation held by the term “recovery” (translated in Romanian as “recuperare,” i.e., “recuperation”): “Therapy includes the psychological side, while recuperation means physical” (Iaco, Interview). Such aspects emphasise the implications and importance of the language of recovery, along with the potential medicalisation of psychological experiences within the psychiatric system.

#### Spiritual healing

Spirituality was a recurring dimension identified in participants’ photographs and narratives that provided a valuable framework for understanding the meanings attributed to recovery within the Romanian cultural context. The religious nuances of recovery from mental distress were eloquently illustrated in a number of accounts reinforcing this: “Recovery means to have a better relationship with God […] He guides me step by step […] I pray to Him, I trust Him … I pray so He will heal me one day” (Emilian, Interview).

Some participants found a fertile ground in the Christian philosophy and church that they perceived as a source of love, (self-)acceptance, growth, and belonging, that facilitated their process of “getting better”. Spiritual resources such as Bible study, praying, and building personal connections with God were crucial steps to finding a “lost” self (“soul”) and coming to terms with their mental health problems. Daniela, for example, discovered her own worth and self-esteem through Christian values and reflection:

Unlike God, I saw myself in a horrible way […] I didn’t appreciate myself and there [at church] I was told that He loves me, and He made me, and if He made me, then I must have some value. (Daniela, Interview)

Praying was revealed as a strategy for distraction from distressful obsessive thoughts by engaging in a meaningful relationship with the divinity that, unlike those around, “listens” to their voice. Praying was also described as an opportunity to express gratitude for recovery:

First of all, I thank God for everything; I thank Him that I am not in a psychiatric hospital where I cannot reason properly; I thank Him that I don’t have dark thoughts [anymore]. (Adriana, Interview)

**Figure 7.**
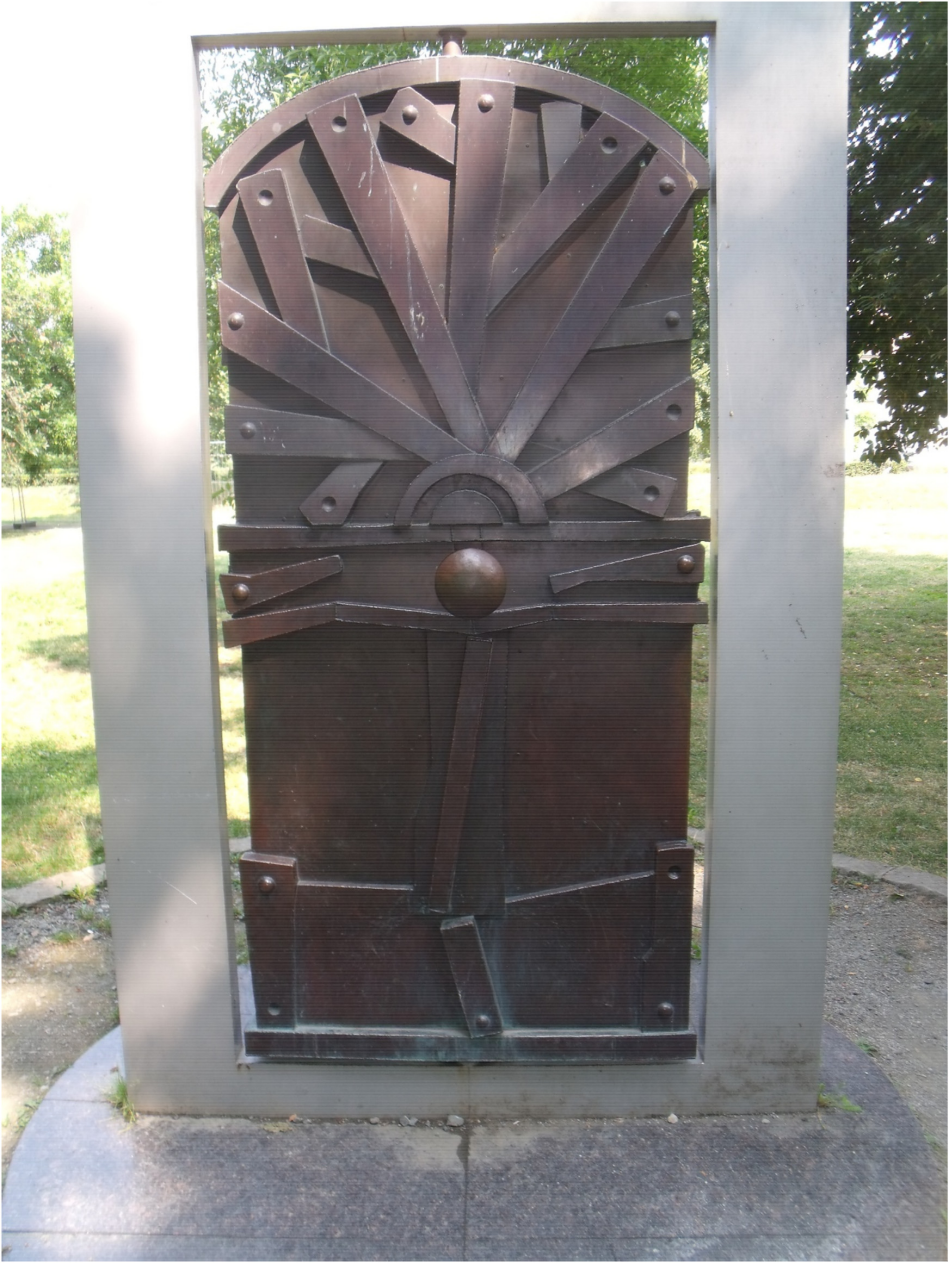
“As a symbol of pain, the cross is ramified into a tree or a multitude of crosses indicating the self-torture imposed by the mental illness.” (Deirdre, Workshop 4).

Such accounts provided examples of positive psychology applied by the research participants most likely from lived experience rather than therapeutic knowledge. Finally, religion was shown to be a source of language and rich metaphors to illustrate the burden and magnitude of living with mental distress, as illustrated in Figure 7.

### Reconstructing life

Recovery was interpreted by most of the participants as a process of reconstructing a life affected by the loss of mental and physical health, relationships, jobs, hope, and education. This life restoration took place on multiple levels: vocationally (“keeping busy”), socially (rebuilding social networks), and existentially (hope for “being well”), in line with their life experiences.

#### Keeping busy

In many instances, recovery was described as an indicator for participants’ levels of functioning or ability to “do things” – from employment to learning new skills and pursuing their hobbies. Employment was particularly important in this context as a source of income to complement their modest pensions, but also for independence and self-esteem:

My job means financial stability, which is important for living an independent life. It helps me rent better accommodation. Also, [it helps] professionally, because it is my first official job […] and when I see that I do the right thing, my self-esteem gets better. (Daniela, Interview)

However, most of the participants were unemployed at the time of conducting this study, which made them reflect on whether they were indeed in recovery. Some participants, in search for a sense of worth and independence, developed their own lucrative business based on their passion for arts (e.g., painting, music) and crafts that they discovered at the day centre: “I make boxes, pencil cases, little things from leather. I also have an income if I manage to sell. It makes me happy because I am useful to my family as well” (Iaco, Interview).

Such occupations were described as therapeutic by participants who also benefited from social exposure which helped them overcome their anxieties and paranoia, but also the stigma associated with their reliance on social benefits. Also therapeutic were the sports, particularly in male participants, who reinforced a sense of routine and self-care through exercise, but also a way to distract themselves from distressing thoughts and feelings. From such accounts, recovery appeared to be embedded in participants’ daily life processes and their tireless search for things to do, with a view of enhancing their life experience and overall quality of life: “Recovery is about finding many possibilities to diversify our life through various stimuli. There are many, many possibilities to enrich our life, develop it” (Deirdre, Workshop 3).

#### Rebuilding social networks

**Figure 8.**
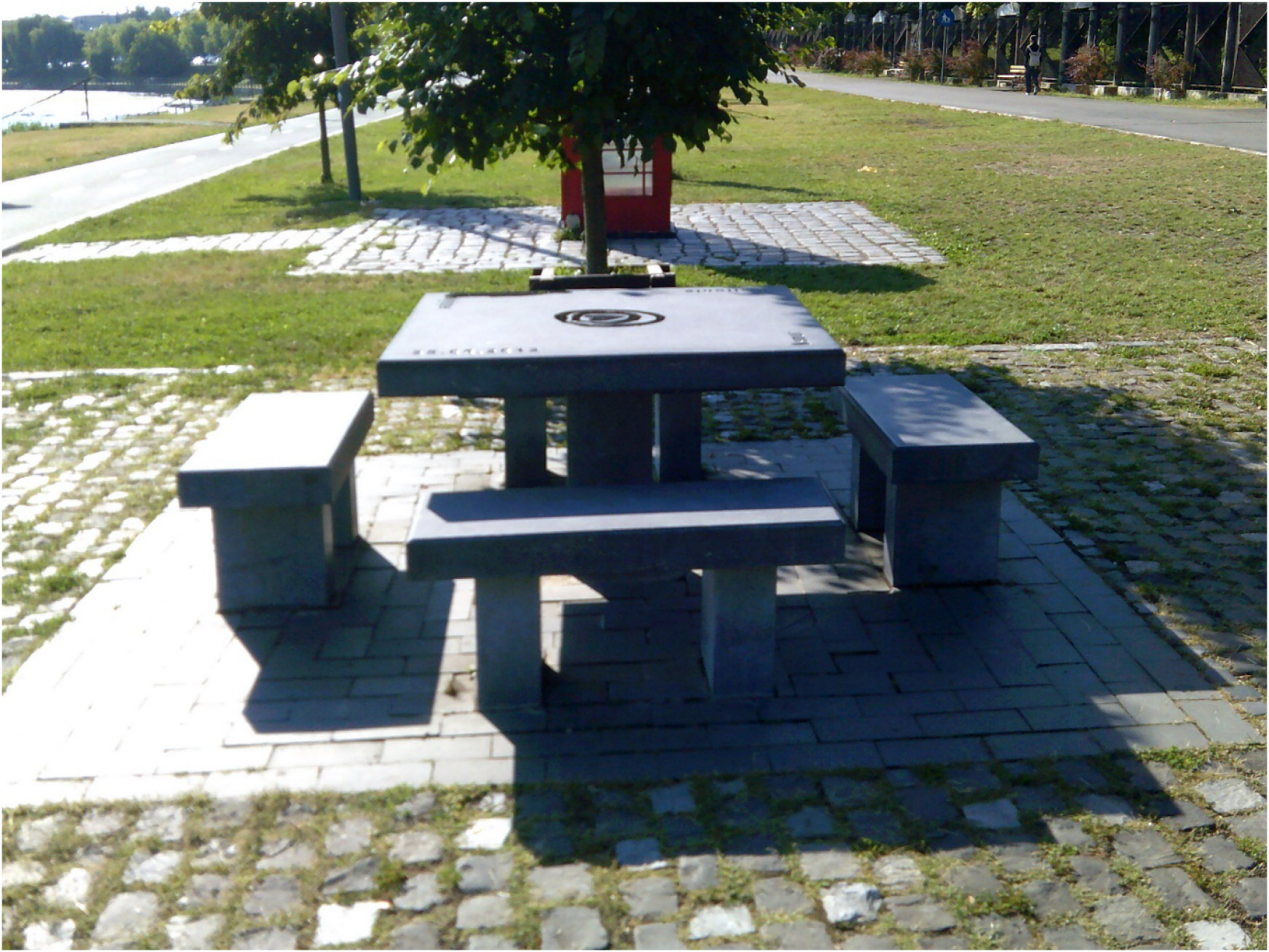
“To recover means to live adequately […] together with other people, belonging, communicating.” (Robert, Interview).

The social dimension of recovery was also prominent in the narratives of research participants who unanimously agreed that recovery did not happen in isolation, but through (re)connection with families, friends, and peers. Therefore, communication was emphasised as key to recovery and developing social skills was conducive to inclusion at a group and community level. To symbolise the importance of communication for recovery, along with feelings of togetherness and belonging that contrasted with social withdrawal that accompanied his mental distress, Robert took a picture of a seating area in his favourite park (Figure 8).

Support from families and friends was considered crucial to recovery, nurtured by feelings of compassion and love unconditioned by their diagnosis and experiences of distress:

My family comes first. My family made me aware somehow that I need a psychiatrist. My kids don’t accept that I am ill, but they love me. They all love me. It's a big step forward when you are loved. (Iaco, Interview)

However, families were described on a spectrum from being supportive and understanding, to being sources of distress adding to the burden of mental illness through conflicts, abuse and trauma, misunderstandings, and control or overprotection leading to a sense of disempowerment: “My parents and I argue sometimes … it's not always good. It affects me a lot because we … argue a lot … it's all that stress that affects me” (Tincuta, Interview).

The role of family in participants’ recovery varied from instrumental (e.g., material and financial support, basic care, administering psychiatric medications, and managing doctors’ appointments) to emotional support and connections that contributed to building a safe environment in which recovery takes place: “I’m blessed to have my family by my side. Being with them gives me a feeling of joy and a psychological balance that helps me recover my life, my energy … to keep afloat, [to be] normal” (Robert, Interview).

### Cultivating hope

Hope was identified in participants’ verbal and visual narratives as an essential ingredient to reconstructing a life impacted by loss: “The final point in life is death [laughs]. But we can also say that it [life] flows to better things in the future. I mean, hope dies last” (Deirdre, Workshop 3). Hope was a dimension interlinked with other themes such as awakening (emerging hope) or healing (hope for getting better), originating in multiple sources reflective of participants’ complex conceptualisation of recovery.

For example, Loredana was spiritually charged with hope through a strong relationship with God; she also expressed hope to maintain her current level of health that allowed her to perform apparently menial daily tasks, but which were nonetheless meaningful to her: “God is rising for us, for us, people … and it charges me with hope. You know, it's not easy to live with this illness without hope” (Loredana, Interview).

For Ioana, hope was associated with life at the day centre, a milestone to her and other participants’ recovery: “The day centre means light … it means hope and good news that it will continue to exist and support people like us” (Ioana, Workshop 4). Hope was also associated by others with improving their mental health (e.g., reduced voices, decreased doses of medication, improved sleep patterns, mood regulation, and more effective anger management).

Finally, some participants related hope to concrete plans for the future, from securing employment and career paths, to personal development, building friendships and even a family of their own, but also migrating one day to a country where they could live a more fulfilling life.

In conclusion, hope was attributed different meanings by different service users who interpreted this concept on an abstract (hope as an idea), emotional (hope as a source of positive feelings), or practical level (hope as personal and professional aspirations in life).

**Figure 9.**
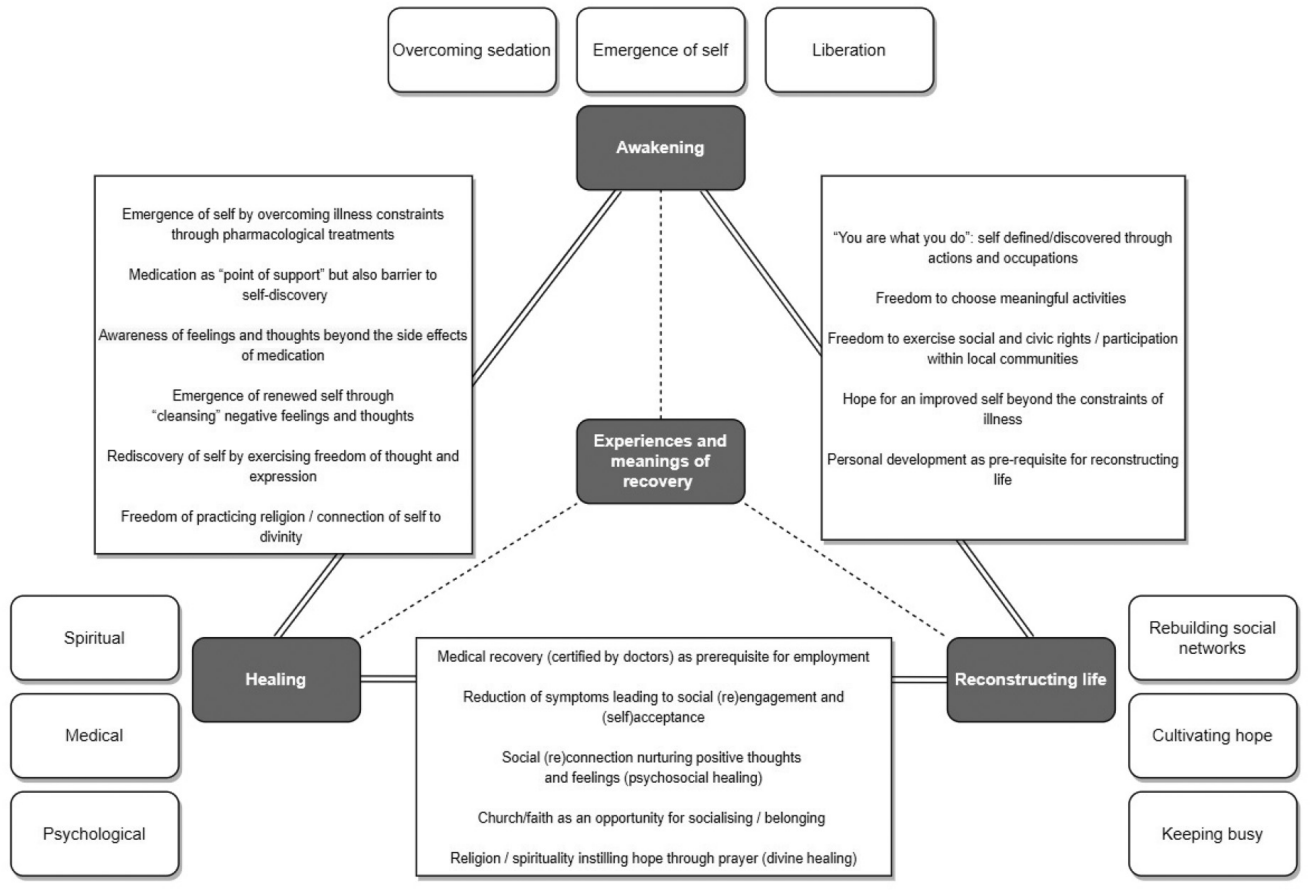
Key themes and subthemes of recovery identified and connections between them.

Themes and subthemes of recovery were enhanced analytically by drawing on complex links developed iteratively at both individual (case-by-case) and group (inter-case analysis) levels, in line with the ideographical approach characteristic to the IPA methodology (Smith et al., 2009) employed by this study (Figure 9). Photography, frequently used by participants as a tool for spatial and semantic exploration, contributed to contextualising lived experiences in various physical and social environments with an impact on recovery. Methodologically, photography also provided a source of triangulation (Glaw et al., 2017) during the data analysis when meanings from textual narratives were corroborated with visual artefacts captured by participants, as illustrated in the visual material included in this article.

## Discussion

This photography-based phenomenological study provides a better understanding of the experience of recovery as lived by a group of Romanian mental health service users. It addresses a research gap in the area of mental health and experiences of recovery in this country where researchers seem to be focused on the perspective of the general public (Neacșu, 2013; Todor, 2013) and psychiatrists (Țebeanu & Macarie, 2013), and less on those living with mental health problems. Understanding what recovery means from the stance of service users is imperative, as suggested by evidence stressing that service users’ expertise, knowledge, and involvement are key to shaping mental health services (Bryant et al., 2011). However, as individuals’ experiences are embedded in, and influenced by, a system of beliefs, customs, values, and ways of life (Bhugra et al., 2021), culture is advanced here as a key dimension that guided the interpretation of data. Furthermore, this prevented the decontextualisation of participants’ experiences from their socio-cultural background.

The meanings attached by the research participants to their recovery through photography were centred around three key aspects: awakening, healing, and reconstructing life, nuanced from a medical, psychological, socio-economic, spiritual, and occasionally political perspective. Their individual and collective stories of recovery indicate the need for a holistic approach to recovery also highlighted by Frost et al. (2017). This contradicts to a certain extent the predominant biomedical approach to their mental health care that was evident in their narratives of “medical healing.”

Recovery as a multi-faceted phenomenon (e.g., clinical, existential, occupational, and psycho-social) was previously identified in various recovery models and frameworks (Leamy et al., 2011; Rapp and Goscha, 2012; Whitley and Drake, 2010). This may suggest a universal understanding of recovery, shared by individuals from various cultures. Hence, it offers the possibility to validate current recovery models in different cultural contexts.

However, a number of particularities of recovery in the Romanian context were highlighted throughout this study. For example, participants contextualised their mental health recovery in the bigger picture of life and existence in a country that was deprived of fundamental human rights for many decades during communism (1947–1989), with an impact on their mental wellbeing. Narratives of traumatic psychiatric treatments (e.g., ECT) intertwined with an oppressive political system were more common in older participants in this study. The narratives of younger participants were contextualised in relation to the challenges faced by post-communist Romania: corruption, unemployment, poverty, and migration, indicating a sense of disenfranchisement. Within this context, social justice frameworks (Carr et al., 2014; Morrow & Weisser, 2012) may guide the recovery work in mental health services in this country by emphasising social, economic, and political factors that facilitate or hinder recovery.

While recovery models originating from English-speaking countries focus on recovery as a personal experience that is unique to each individual (Leamy et al., 2011), this study emphasised both the individual and the collectivistic contexts of recovery. For example, recovery as awakening does not take place at personal and inter-personal levels only, but also at the confluence between personal life and the cultural, historic, and socio-political circumstances.

In some cases, participants’ recovery seemed to follow a journey interlinked with the historical trajectory of their country from oppression to freedom historically marked by the Romanian Revolution in 1989 (Siani-Davies, 2007) to which participants frequently referred in their “liberation” narratives.

Psychiatric treatments and practices during and after communism gave their narratives an authentic flavour that enhanced the understanding of meanings attributed to recovery through a lens of oppression. For some participants, oppression was linked to their traumatic experiences and abuse in their family, which triggered or added to their distress. A sense of prescriptiveness in terms of feeling pressured to “normalise” their behaviours according to family, psychiatry, and societal standards was noted, evident in the awakening/liberation theme. Nonetheless, positive experiences of family support, psychiatric care, and community involvement were also recorded. However, such positive encounters appeared to be an exception, which may explain the low levels of empowerment, i.e., choice and control over their lives and mental health, generally reflected in participants’ narratives. Therefore, a better understanding of the family context and processes that are known to be determined culturally (Bornstein & Lansford, 2019) is claimed here to be key to recovery work.

The lack of understanding of psycho-social and cultural aspects may be problematic for implementing person-centred and recovery-oriented mental health strategies in Romania. This in an issue explored by Matza (2018) in his account of post-socialist Russia transitioning from a biomedical materialist tradition that dominated the psychiatric system to a revitalisation of psychotherapeutic approaches. Unlike the boom of psychologically oriented therapies in Russia described by Matza (2018), there is a real risk that mental health services in Romania reinforce a reductionist perspective to treatment if their focus continues to be solely on the clinical recovery, as portrayed by the research participants. Some solutions are suggested by the cultural findings of this study, e.g., spirituality, which was unsurprising considering that more than 90% of the Romanian population is Christian-Orthodox. Spirituality was a recurrent theme of recovery verbalised by more than half of the participants in this study in the form of religious practices such as praying, going to church, and Bible study. Similar to research conducted in post-soviet Russia (Zigon, 2011), such religious practices provided participants with a sense of moral awareness and self-development, and supported their discovery of renewed, ethically “good” identities (personhood) previously “spoiled” by mental illness. On the same note, some participants considered local faith leaders, instead of mental health professionals, as the first point of call for counselling and support when in distress. As a result, spirituality was suggested by participants as a complementary route of “healing” along with medical and psychological treatments, which is supported by Chidarikire (2012). Zigon (2011) also highlights the complementarity between therapeutic models and church-run rehabilitation programmes focused on self-transformation, that may inform the mental health reform in Romania.

Although emphasised in numerous studies worldwide (Fallot, 2007; Ho et al., 2016), spirituality is not generally acknowledged in recovery models (Rapp and Goscha, 2012; Whitley and Drake, 2010) that refer to communities from a generic social perspective instead of spiritual or religious. However, as an exception, the CHIME recovery framework (Leamy et al., 2011) emphasised the role of spirituality and belonging to a faith community in the recovery of Black and Minority Ethnic (BME) individuals.

In light of this, the responsibility for recovery needs to be shared collaboratively between individuals, mental health professionals, faith leaders, and governmental bodies. The expanse of neoliberal ideas and policies in post-communist Romania emphasising individual roles and responsibility as opposed to a socialist collective approach (Ban, 2016) may pose some challenges and increase the pressure on personal recovery. However, a collaborative strategy to recovery can be facilitated through appropriate policies that acknowledge the impact of political and socio-economic factors on mental distress and recovery, along with cultural determinants.

On this note, one participant (Deirdre) referred to recovery as a process that involves “awakening” as activism, with service users (“an army of foot soldiers”) ready to march the streets to “make noise” (i.e., protest, make their voices heard), and become more engaged in terms of civic responsibility and social participation. Although timidly expressed, this view portrays the latent potential of the Romanian service users to voice their concerns and seek representation outside the walls of their day centre which remains, nonetheless, the cornerstone of recovery for most of them. While the benefits of the community mental health centre were evident in participants’ narratives and photographs, the long history of accessing services at the day centre (more than twenty years in some cases) raised questions around the risk of re-institutionalisation and its impact on recovery (i.e., participants becoming dependent on community services). This may be related to participants’ status as “beneficiaries” (Rom. “beneficiari”) of mental health services that risks portraying them as passive receivers, consumers, or “idlers,” as expressed by some research participants, rather than contributors to their communities. This language risks reinforcing stigma and disempowerment (Richards, 2018), particularly in a society in which echoes of the communist work ethic and social undesirability of idleness and social parasitism persist (Heintz, 2008). Furthermore, it does not reflect the full extent of participants’ experiences and their aim to reconstruct their lives in the context of family, local community, and wider society. It was revealed in this study that participants are invaluable sources of creative potential and skills from which local communities could greatly benefit while empowering those living with mental health problems and, consequently, supporting their recovery. Denying people with mental health problems the opportunity to contribute to the cultural, social, and economic life of their communities will undoubtedly result in further marginalising and disempowering them.

In conclusion, although recovery is reinforced in this study as a complex, multi-faceted, and non-linear process, several particularities suggest that a full understanding of recovery processes cannot be divorced from the socio-economic, political, and cultural context. As a consequence, importing recovery models to a culturally diverse population may not be a straightforward process for the Romanian stakeholders, service providers, and policymakers. In light of participants’ interpretation of recovery as a restoration of positive feelings and thoughts or psychological “healing,” the lack of psychotherapeutic services in Romania, for instance, raises some concerns in relation to the effectiveness of the mental health reform and its congruence with service users’ views and conceptualisation of recovery.

### Limitations

This study, as with other qualitative research, may be limited in terms of generalising the findings to the Romanian population, although generalisation is not necessarily the goal of qualitative research (Polit & Beck, 2010). Furthermore, the research participants were limited to service users from the community, not inpatient settings. This may have impacted the richness of perspectives that could have provided an additional layer of understanding by potentially revealing different views on recovery. Language was another limitation, mainly in relation to the lack of an equivalent term for “recovery” in Romanian. The closest term identified by the researcher was “recuperation” (Rom. “recuperare”), which has medical connotations, therefore participants’ view was potentially biased towards clinical recovery. However, the language was negotiated throughout the project with new terminologies advanced by the research participants (“healing” of psychological “wounds”; “mending fractured personalities”; “awakening” of senses, motivation, and self). Photographic metaphors and the phenomenological approach aided with addressing this linguistic gap that did not significantly impact the translation and interpretation of transcripts. Finally, this study captured only a “snapshot” of recovery that reflects individual experiences and perspectives on the researched phenomenon during a relatively short period of time (approximately two months). On reflection, the employment of an ethnographic design could have partly addressed this issue, and may have offered a better vantage point on processes of recovery throughout a longer period of time.

### Directions for future research

This study adds to the international body of literature on mental health, recovery, and culture (Gamieldien et al., 2020; Gopalkrishnan, 2018; Myers, 2010) by exploring experiences of recovery through a socio-economic, cultural, political, and historical lens. The use of photography enhanced the phenomenological design of this study by eliciting consciousness processes that may have not been possible through conventional interviews relying on spoken language only (Milasan et al., 2020). It also gave participants the freedom to visually explore physical spaces with pronounced cultural connotations (e.g., homes, churches, parks, and hospitals) that supported their verbal narratives. Alternative approaches such as digital storytelling (Colucci & McDonough, 2019), videography (Petros et al., 2016), and participatory arts (Stickley et al., 2018) may also be considered in future recovery studies. From a methodological and conceptual point of view, this study may serve as a blueprint for cultural studies conducted in other former communist countries in Eastern Europe, with a view to developing comparative perspectives on recovery and its cultural interpretation.

### Implications for practice

Understanding the experience and meanings of recovery from the perspective of Romanian mental health service users is the first step to developing culturally sensitive and person-centred recovery pathways in this country. Furthermore, the findings of this study can inform the development of comprehensive recovery plans that currently seem to have a pronounced biomedical focus. Challenging the predominant clinical focus of psychiatry in Romania and promoting a holistic approach to recovery may also benefit the mental health reform in this country. For example, the need for therapy to support psychological “healing,” vocational support towards gaining employment, and a stronger emphasis on spirituality as a key resource in recovery are only a few areas that need to be considered as an alternative to, or complementary with, psychiatric treatments. Additionally, facilitating social participation and involvement of service users in policy and service development is key to shaping services that are true to their complex needs and understanding. Finally, the results of this study reiterate the idea that the historical, political, and spiritual dimensions of recovery constitute key cultural dimensions that need to be factored into the development of mental health services.

## Conclusion

This photography-based phenomenological study is the first to explore lived experiences of recovery as perceived and interpreted by Romanian mental health service users. The findings reflect the depth and complexity of this phenomenon that exceed the realm of psychiatry and place recovery on psychological, social, existential, occupational, and spiritual coordinates. Additionally, recovery is shown to be culturally, socially, politically, and historically determined. Without a comprehensive exploration at these levels, the understanding of the experience of recovery may be limited to the knowledge provided by the current recovery models whose applicability to various cultural contexts remains uncertain.

## References

[bibr1-13634615221119373] Andronic A. O. Andronic R. L. (2017). Community-based mental health services in Romania. Scientific Research and Education in the Air Force, 19(2), 19–22. 10.19062/2247-3173.2017.19.2.2

[bibr2-13634615221119373] Anthony W. A. (1993). Recovery from mental illness: The guiding vision of the mental health service system in the 1990s. Psychosocial Rehabilitation Journal, 16(4), 11–23. 10.1037/h0095655

[bibr4-13634615221119373] Ban C. (2016). *Ruling ideas: How global neoliberalism goes local*. Oxford University Press.

[bibr5-13634615221119373] Barker P. J. Buchanan-Barker P. (2005). The Tidal Model: A guide for mental health professionals. Brunner-Routledge.

[bibr6-13634615221119373] Bhugra D. Watson C. Wijesuriya R. (2021). Culture and mental illnesses, International Review of Psychiatry, 33(1), 1–2. 10.1080/09540261.2020.1777748 33599563

[bibr7-13634615221119373] Bornstein M. H. Lansford J. E. (2019). Culture and family functioning. In Fiese B. H. Celano M. Deater-Deckard K. Jouriles E. N. Whisman M. A. (Eds.), APA Handbook of contemporary family psychology: Applications and broad impact of family psychology (pp. 417–436). American Psychological Association.

[bibr8-13634615221119373] Boucher M.-E. Groleau D. Whitley R. (2019). Recovery from severe mental illness in Québec: The role of culture and place. Health & Place, 56(1), 63–69. 10.1016/j.healthplace.2019.01.008 30710835

[bibr9-13634615221119373] Bracke P. Delaruelle K. Verhaeghe M. (2019). Dominant cultural and personal stigma beliefs and the utilization of mental health services: A cross-national comparison. Frontiers in Sociology, 4(40), 1–12. 10.3389/fsoc.2019.00040 33869363 PMC8022809

[bibr10-13634615221119373] Bryant W. Tibbs A. Clark J. (2011). Visualising a safe space: The perspective of people using mental health day services. Disability and Society, 26(5), 611–628. 10.1080/09687599.2011.589194

[bibr11-13634615221119373] Cabassa L. J. Nicasio A. Whitley R. (2013). Picturing recovery: A photovoice exploration of recovery dimensions among people with serious mental illness. Psychiatry Services, 64(9), 1–11. 10.1176/appi.ps.201200503 PMC3864368 23728528

[bibr12-13634615221119373] Çam M. O. Uğuryol M. (2019). From mental disorder to recovery: Cultural effect. Current Approaches in Psychiatry, 11(1), 55–64. 10.18863/pgy.391783

[bibr13-13634615221119373] Carr E. R. Bhagwat R. Miller R. Ponce A. N. (2014). Training in mental health recovery and social justice in the public sector. The Counseling Psychologist, 42(8), 1108–1135. 10.1177/0011000014555200

[bibr14-13634615221119373] Chidarikire S. (2012). Spirituality: The neglected dimension of holistic mental health care. Advances in Mental Health, 10(3), 298–302. 10.5172/jamh.2012.10.3.298

[bibr15-13634615221119373] Chiu M. Ho W. Lo W. Yiu M. (2010). Operationalization of the SAMHSA model of recovery: A quality of life perspective. International Journal of Quality of Life Aspects of Treatment, Care and Rehabilitation, 19(1), 1–13. 10.1007/s11136-009-9555-2 19921548

[bibr16-13634615221119373] Colucci E. McDonough S. (2019). Recovering from mental illness and suicidal behaviour in a culturally diverse context. In Lo V. Berry C. Liping G. (Eds.), Film and the Chinese medical humanities (pp. 205–225). Routledge.

[bibr17-13634615221119373] Deacon B. J. (2013). The biomedical model of mental disorder: A critical analysis of its validity, utility, and effects on psychotherapy research. Clinical Psychology Review, 33(7), 846–861. 10.1016/j.cpr.2012.09.007 23664634

[bibr18-13634615221119373] Denzin N. K. (2004). Symbolic interactionism. In Flick U. von Kardorff E. Steinke I. (Eds.), A companion to qualitative research (pp. 81–87). Sage Publications Ltd.

[bibr19-13634615221119373] Ellis C. Flaherty M. G. (1992). Investigating subjectivity: Research on lived experience. Sage Publications, Inc.

[bibr20-13634615221119373] Eltaiba N. Harries M. (2015). Reflections on recovery in mental health: Perspectives from a Muslim culture. Social Work in Health Care, 54(8), 725–737. 10.1080/00981389.2015.1046574 26399491

[bibr21-13634615221119373] Fallot R. D. (2007). Spirituality and religion in recovery: Some current issues. Psychiatric Rehabilitation Journal, 30(4), 261–270. 10.2975/30.4.2007.261.270 17458450

[bibr22-13634615221119373] Friedman J. R. (2016). “A world crazier than us”: Vanishing social contexts and the consequences for psychiatric practice in contemporary Romania. Transcultural Psychiatry, 53(2), 176–197. 10.1177/1363461515590917 26134545

[bibr23-13634615221119373] Frost B. G. Tirupati S. Johnston S. , et al. (2017). An integrated recovery-oriented model (IRM) for mental health services: Evolution and challenges. BMC Psychiatry, 17(22), 1–7. 10.1186/s12888-016-1164-3 28095811 PMC5240195

[bibr24-13634615221119373] Gamieldien F. Galvaan R. Myers B. Sorsdahl K. (2020). Exploration of recovery of people living with severe mental illness (SMI) in low-income and middle-income countries (LMIC): A scoping review protocol. BMJ Open, 10, e032912. 10.1136/bmjopen-2019-032912 PMC7044907 32019817

[bibr25-13634615221119373] Glaw X. Inder K. Kable A. Hazelton M. (2017). Visual methodologies in qualitative research: Autophotography and photo elicitation applied to mental health research. International Journal of Qualitative Methods, 16(1), 1–8. 10.1177/1609406917748215

[bibr26-13634615221119373] Gopal S. Henderson A. R. (2015). Trans-cultural study of recovery from severe enduring mental illness in Chennai, India and Perth, Western Australia. Journal of Psychosocial Rehabilitation and Mental Health, 2(1), 51–57. 10.1007/s40737-015-0031-8

[bibr27-13634615221119373] Gopalkrishnan N. (2018). Cultural diversity and mental health: Considerations for policy and practice. Frontiers in Public Health, 6, 179. 10.3389/fpubh.2018.00179 29971226 PMC6018386

[bibr28-13634615221119373] Heintz M. (2008). Changes in work ethic in Eastern Europe: The case of Romania. Autrepart, 4(48), 45–57. 10.3917/autr.048.0045

[bibr29-13634615221119373] Ho R. T. Chan C. K. Lo P. H. Wong P. H. Chan C. L. Leung P. P. Chen E. Y. (2016). Understandings of spirituality and its role in illness recovery in persons with schizophrenia and mental-health professionals: A qualitative study. BMC Psychiatry, 16(86), 1–11. 10.1186/s12888-016-0796-7 27038910 PMC4818963

[bibr30-13634615221119373] Lapsley H. Nikora L. W. Black R. (2002). “*Kia Mauri Tau!”: Narratives of recovery from disabling mental health problems*. Report of the University of Waikato Mental Health Narratives Project. Wellington, New Zealand: Mental Health Commission.

[bibr31-13634615221119373] Law H. Morrison A. P. (2014). Recovery in psychosis: A Delphi study with experts by experience. Schizophrenia Bulletin, 40(6), 1347–1355. 10.1093/schbul/sbu047 24727194 PMC4193718

[bibr32-13634615221119373] Leamy M. Bird V. Le Boutillier C. Williams J. Slade M. (2011). Conceptual framework for personal recovery in mental health: Systematic review and narrative synthesis. The British Journal of Psychiatry: The Journal of Mental Science, 199(6), 445–452. 10.1192/bjp.bp.110.083733 22130746

[bibr33-13634615221119373] Macpherson R. Pesola F. Leamy M. Bird V. Le Boutillier C. Williams J. Slade M. (2016). The relationship between clinical and recovery dimensions of outcome in mental health. Schizophrenia Research, 175(1–3), 142–147. 10.1016/j.schres.2015.10.031 26527245

[bibr34-13634615221119373] Matza T. (2018). Shock Therapy: Psychology, Precarity, and Well-Being in Postsocialist Russia. Duke University Press.

[bibr68-13634615221119373] Milasan L. H. (2022). Photo-elicitation: Unleashing imagery in healthcare research. In Hinsliff-Smith, K., McGarry, J., & Ali, P. (Eds.), Arts based health care research: A multidisciplinary perspective, (pp. 51–67). Springer.

[bibr35-13634615221119373] Milasan L. H. Bingley A. F. Fisher N. R. (2020). The big picture of recovery: A systematic review on the evidence of photography-based methods in researching recovery from mental distress. Arts & Health, 14(2), 165–185. 10.1080/17533015.2020.1855453 33252304

[bibr36-13634615221119373] Mizock L. Russinova Z. Shani R. (2014). New roads paved on losses: Photovoice perspectives about recovery from mental illness. Qualitative Health Research, 24(11), 1481–1491. 10.1177/1049732314548686 25168704

[bibr37-13634615221119373] Morrow M. Weisser J. (2012). Towards a social justice framework of mental health recovery. Studies in Social Justice, 6(1), 27–43. 10.26522/ssj.v6i1.1067

[bibr38-13634615221119373] Myers N. L. (2010). Culture, stress and recovery from schizophrenia: Lessons from the field for global mental health. Culture, Medicine and Psychiatry, 34(3), 500–528. 10.1007/s11013-010-9186-7 20571905 PMC3068598

[bibr39-13634615221119373] Neacșu D. (2013). Public understanding of mental illness: Results from a Romanian sample. Journal of Experiential Psychotherapy, 16(4), 30–35.

[bibr40-13634615221119373] Ng R. M. K. Pearson V. Lam M. Law C. W. Chiu C. P. Y. Chen E. Y. H. (2008). What does recovery from schizophrenia mean? Perceptions of Long-Term Patients. International Journal of Social Psychiatry, 54(2), 118–130. 10.1177/0020764007084600 18488406

[bibr41-13634615221119373] Nørreklit L. (2006). The double hermeneutics of life world: A perspective on the social, dialogue and interpretation. Institut for Uddannelse, Læring og Filosofi, Aalborg University. https://www.forskningsdatabasen.dk/en/catalog/2389382675

[bibr42-13634615221119373] O’Hagan M. (2001). Recovery competencies for New Zealand mental health workers. Mental Health Commission. https://files.eric.ed.gov/fulltext/ED457512.pdf

[bibr43-13634615221119373] Petros R. Solomon P. Linz S. DeCesaris M. Hanrahan N. P. (2016). Autovideography: The lived experience of recovery for adults with serious mental illness. Psychiatric Quarterly, 87(3), 417–426. https://doi.org/10.1007/s11126-015-9397-8 26506921 10.1007/s11126-015-9397-8

[bibr44-13634615221119373] Phelps J. Nadim M. (2014). Ethnicity: Overview. In Teo T. (Ed.), Encyclopaedia of critical psychology (pp. 1231–1237). Springer.

[bibr45-13634615221119373] Polit D. F. Beck C. T. (2010). Generalization in quantitative and qualitative research: Myths and strategies. International Journal of Nursing Studies, 47(11), 1451–1458. 10.1016/j.ijnurstu.2010.06.004 20598692

[bibr46-13634615221119373] Price-Robertson R. Obradovic A. Morgan B. (2016). Relational recovery: Beyond individualism in the recovery approach. Advances in Mental Health, 15(2), 1–13. 10.1080/18387357.2016.1243014

[bibr47-13634615221119373] Rapp C. A. Goscha R. J. (2012). The strengths model: A recovery-oriented approach to mental health services (3rd ed.). Oxford University Press.

[bibr48-13634615221119373] Recovery in the Bin. (2017). *10 key principles of Recovery in the Bin*. https://recoveryinthebin.org/ritbkeyprinciples/

[bibr49-13634615221119373] Richards V. (2018). The importance of language in mental health care. The Lancet (Psychiatry), 5(6), 460–461. 10.1016/S2215-0366(18)30042-7 29482994

[bibr50-13634615221119373] Rudnick A. (2012). Recovery of people with mental illness: Philosophical and related perspectives. Oxford University Press.

[bibr69-13634615221119373] Schwartz T. (1992). Anthropology and psychology: An unrequited relationship. In Schwartz, T., White, G., & Lutz, C. (Eds.), New directions in psychological anthropology (pp. 324–349). Cambridge University Press.

[bibr51-13634615221119373] Shields-Zeeman L. Petrea I. Smit F. Hipple-Walters B. Dedovic J. Rojnic-Kuzman M. , et al. (2020). Towards community-based and recovery-oriented care for severe mental disorders in southern and Eastern Europe: Aims and design of a multi-country implementation and evaluation study (RECOVER-E). International Journal of Mental Health Systems, 14(30), 1–14. 10.1186/s13033-020-00361-y 32336984 PMC7178587

[bibr52-13634615221119373] Siani-Davies P. (2007). The Romanian Revolution of December 1989. Cornell University Press.

[bibr53-13634615221119373] Sitvast J. (2015). Recovery in mental health care with the aid of photo-stories: An action research based on the principles of hermeneutic photography. Nursing and Health, 3(6), 139–146. 10.13189/nh.2015.030602

[bibr54-13634615221119373] Slade M. (2009). Personal recovery and mental illness: A guide for mental health professionals. Cambridge University Press.

[bibr55-13634615221119373] Slade M. Amering M. Farkas M. Hamilton B. O’Hagan M. Panther G. Perkins R. Shepherd G. Tse S. Whitley R. (2014). Uses and abuses of recovery: Implementing recovery-oriented practices in mental health systems. World Psychiatry, 13(1), 12–20. https://doi.org/10.1002/wps.20084 24497237 10.1002/wps.20084 PMC3918008

[bibr56-13634615221119373] Slade M. Wallace G. (2017). Recovery and mental health. In Slade M. Oades L. Jarden A. (Eds.), Wellbeing, recovery and mental health (pp. 24–34). Cambridge University Press.

[bibr57-13634615221119373] Smith J. A. Flowers P. Larkin M. (2009). Interpretative phenomenological analysis: Theory, method and research. Sage Publications Ltd.

[bibr58-13634615221119373] Stickley T. Wright N. Slade M. (2018). The art of recovery: Outcomes from participatory arts activities for people using mental health services. Journal of Mental Health, 27(4), 367–373. 10.1080/09638237.2018.143760 29447483

[bibr59-13634615221119373] Țebeanu A. V. Macarie G. F. (2013). The role of education in mental health: Considerations of professionals from a psychiatric clinic regarding its implications in the process of community integration for former patients. Procedia - Social and Behavioral Sciences, 76, 827–831. 10.1016/j.sbspro.2013.04.214

[bibr60-13634615221119373] Thara R. (2012). Consumer perceptions of recovery: An Indian perspective. World Psychiatry, 11(3), 169–170. 10.1002/j.2051-5545.2012.tb00125.x 23024675 PMC3449346

[bibr61-13634615221119373] Todor I. (2013). Opinions about mental illness. Procedia – Social and Behavioral Sciences, 82(1), 209–214. 10.1016/j.sbspro.2013.06.247

[bibr62-13634615221119373] Tse S. Tang J. Kan A. (2012). Patient involvement in mental health care: Culture, communication and caution. Health Expectations, 18(1), 3–7. 10.1111/hex.12014 23067250 PMC5060755

[bibr63-13634615221119373] Vansteenkiste T. Morrens M. Westerhof G. J. (2021). Images of recovery: A PhotoVoice study on visual narratives of personal recovery in persons with serious mental illness. Community Mental Health Journal, 57(1), 151–1163. 10.1007/s10597-020-00746-w 33230705

[bibr64-13634615221119373] Whitley R. Drake R. E. (2010). Recovery: A dimensional approach. Psychiatric Services (Washington, D.C.), 61(12), 1248–1250. 10.1176/ps.2010.61.12.1248 21123410

[bibr65-13634615221119373] World Health Organization. (2017). *Culture and reform of mental health care in Central and Eastern Europe*. http://www.euro.who.int/en/publications/abstracts/culture-and-reform-ofmental-health-care-in-central-and-eastern-europe-2018

[bibr66-13634615221119373] Zigon J. (2011). A moral and ethical assemblage in Russian orthodox drug rehabilitation. Ethos (Berkeley, Calif), 39(1), 30–50. https://doi.org/10.1111/j.1548-1352.2010.01169.x

